# The Administration of *Escherichia coli* Nissle 1917 Ameliorates Development of DSS-Induced Colitis in Mice

**DOI:** 10.3389/fphar.2018.00468

**Published:** 2018-05-11

**Authors:** Alba Rodríguez-Nogales, Francesca Algieri, José Garrido-Mesa, Teresa Vezza, Maria P. Utrilla, Natalia Chueca, Jose A. Fernández-Caballero, Federico García, Maria E. Rodríguez-Cabezas, Julio Gálvez

**Affiliations:** ^1^CIBEREHD, Department of Pharmacology, Instituto de Investigación Biosanitaria de Granada, Centre for Biomedical Research (CIBM), University of Granada, Granada, Spain; ^2^Department of Microbiology, Complejo Hospitalario Universitario de Granada, Instituto de Investigación Biosanitaria de Granada, Granada, Spain

**Keywords:** probiotic, microRNA, pyrosequencing, intestinal microbiota, DSS colitis

## Abstract

The beneficial effects of probiotics on immune-based pathologies such as inflammatory bowel disease (IBD) have been well reported. However, their exact mechanisms have not been fully elucidated. Few studies have focused on the impact of probiotics on the composition of the colonic microbiota. The aim of the present study was to correlate the intestinal anti-inflammatory activity of the probiotic *Escherichia coli* Nissle 1917 (EcN) in the dextran sodium sulfate (DSS) model of mouse colitis with the changes induced in colonic microbiota populations. EcN prevented the DSS-induced colonic damage, as evidenced by lower disease activity index (DAI) values and colonic weight/length ratio, when compared with untreated control mice. The beneficial effects were confirmed biochemically, since the probiotic treatment improved the colonic expression of different cytokines and proteins involved in epithelial integrity. In addition, it restored the expression of different micro-RNAs (miR-143, miR-150, miR-155, miR-223, and miR-375) involved in the inflammatory response that occurs in colitic mice. Finally, the characterization of the colonic microbiota by pyrosequencing showed that the probiotic administration was able to counteract the dysbiosis associated with the intestinal inflammatory process. This effect was evidenced by an increase in bacterial diversity in comparison with untreated colitic mice. The intestinal anti-inflammatory effects of the probiotic EcN were associated with an amelioration of the altered gut microbiome in mouse experimental colitis, especially when considering bacterial diversity, which is reduced in these intestinal conditions. Moreover, this probiotic has shown an ability to modulate expression levels of miRNAs and different mediators of the immune response involved in gut inflammation. This modulation could also be of great interest to understand the mechanism of action of this probiotic in the treatment of IBD.

## Introduction

The gut microbiota is a very complex ecosystem that establishes a symbiotic relationship with the host at the intestinal epithelium. Although bacteria are considered the most prominent component, gut microbiota also includes a few eukaryotic fungi, viruses, and some Archaea (Power et al., 2014). Microbiota composition changes along the axis of the human gastrointestinal tract, and surface-adherent and luminal microbial populations could differ from each other (Eckburg et al., 2005). This particular bacterial composition is essential for maintaining the structural integrity of the gut mucosal barrier, immunomodulation, and nutrient and xenobiotic metabolism, among others’ effects. Moreover, the microbiota helps the host to distinguish commensal organisms from episodic pathogens to orchestrate the adequate responses (Patel and Lin, 2010). However, when the composition of the microbiota is impaired (dysbiosis), this symbiotic relationship does not function properly and the host may identify the normal microbiota constituents as harmful, which could trigger an inappropriate immune response. This alteration could ultimately lead to inflammatory conditions such as IBD. Different mediators, such as miRNAs, are involved in the build-up of the inflammatory response in these conditions. Some miRNAs have been described to participate in the control of strategic cellular functions in the immune system, including differentiation, proliferation, signal transduction, and apoptosis (Xu and Zhang, 2016). Furthermore, miR-21, miR-122a, miR-155, and miR-150 have been associated with impairment of tight junction proteins and increased intestinal epithelial permeability (Bian et al., 2011; Ye et al., 2011).

Therefore, the therapeutic approaches designed to modify and restore the intestinal microbiota composition and the inflammatory response could be of clinical relevance. Among these treatments, the administration of probiotics, defined as “live microorganisms, which, when consumed in adequate amounts, confer a health benefit on the host” by an Expert Consultation Committee at a meeting convened by the Food and Agriculture Organization/World Health Organization in 2001, may play a prominent role in the clinical management of these intestinal conditions. Several mechanisms of action have been proposed to explain their beneficial effects, including enhancement of the epithelial barrier function, increased adhesion to the intestinal mucosa, and concomitant inhibition of pathogen adhesion, as well as competitive exclusion of pathogenic microorganisms through production of antimicrobial substances and modulation of the immune system (Prisciandaro et al., 2009). However, not all microorganisms have the same efficacy in the treatment of these conditions or display the same properties. These differences are explained by their heterogeneity. In fact, only a few clinical trials have been successful, despite a high number of studies describing the efficacy of probiotics in experimental models of colitis have been conducted (Ghouri et al., 2014). Human trials have been performed either with bacterial mixtures, like VSL#3, or with single probiotics, like *Lactobacillus rhamnosus* GG, *Saccharomyces boulardii*, or EcN. Although the initial trials showed benefits with all these probiotics, subsequent studies or systematic reviews have questioned their efficacy, reporting variations in probiotics effectiveness depending on the form of IBD evaluated (UC, CD, or pouchitis) or the stage (exacerbation or remission) of the intestinal conditions when the probiotics were administered (Singh et al., 2015). In this regard, when considering EcN, it was shown that despite the administration of this probiotic in conjunction with standard therapy to UC patients did not increase the induction rate of remission, the treatment with this probiotic could have a similar effect to mesalazine in maintaining the remission (Kruis et al., 2004). In fact, since 2011, the “American Recommendations for probiotic use” give very strong “A” recommendations for the use of EcN for the maintenance of remission in human UC (Floch et al., 2011). Several studies have reported immuno-modulatory properties for EcN through different mechanisms that could be responsible for its clinical efficacy in IBD. These mechanisms are mainly associated with the ability of this probiotic to modulate the cytokine production in immune cells interfering with different cell pathways, including those involving different transcription factors like NF-κB and modulating the activity of the MAPKs (Grabig et al., 2006; Hafez et al., 2010). Moreover, the antimicrobial properties of this probiotic, which comprise both a direct effect and the stimulation of defensins production by intestinal epithelial cells, can also contribute to its beneficial effects in experimental colitis as well as in human IBD (Wehkamp et al., 2004). However, despite numerous studies have described the beneficial effects of different probiotics in experimental colitis, they have not elucidated how probiotics affect the microbiota composition in these conditions, where the dysbiosis has been demonstrated. Therefore, the aim of the present study was to investigate the effects of the administration of EcN in an experimental model of colitis in mice, evaluating its impact on the microbiota composition and correlating this to the immuno-modulatory properties of this probiotic.

## Materials and Methods

### Reagents and Probiotics

All chemicals were obtained from Sigma-Aldrich Quimica (Madrid, Spain), unless otherwise stated. EcN was provided by Ardeypharm GmbH (Herdecke, Germany).

### Dextran Sodium Sulfate (DSS)-Induced Colitis Mouse Model

The animals were housed in Makrolon cages, maintained in an air-conditioned atmosphere with a 12 h light-dark cycle, and provided with free access to tap water and food. All studies were conducted according to the ‘Guide for the Care and Use of Laboratory Animals’ as promulgated by the National Institute of Health and the protocols approved by the “Animal Experimentation Ethics Committee” of the University of Granada (Spain; Ref. No. CEEA-2010-286).

**FIGURE 1:**
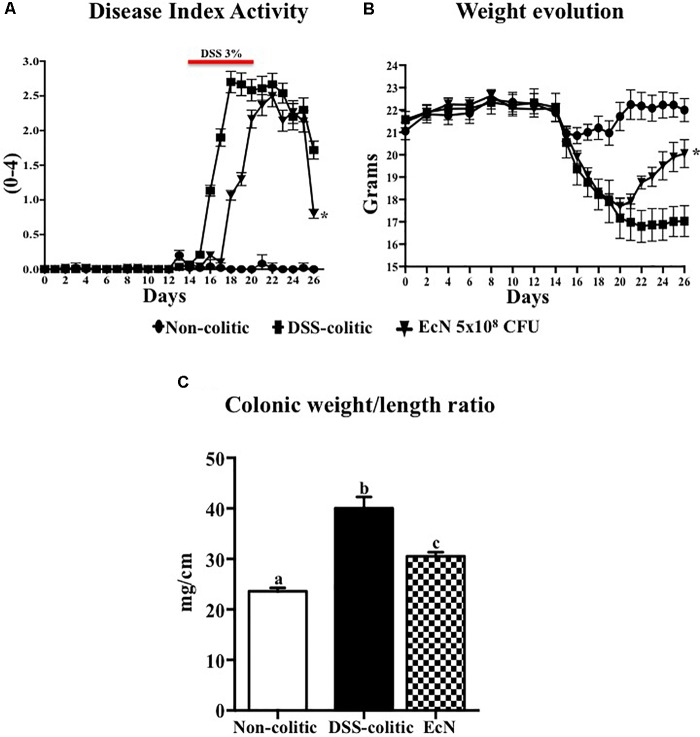
Effect of *Escherichia coli* Nissle 1917 (EcN) in DSS-induced colitis model. **(A,B)** Disease activity index (DAI) values and weight evolution in DSS-colitic mice over the 26-day experimental period. Statistical significance among groups was evaluated by one-way ANOVA followed by Dunnett’s test. ^∗^*p* < 0.05 vs. DSS-colitic group. **(C)** Colonic weight/length ratio, expressed as means ± SEM. Non-colitic group (*n* = 10), DSS-colitic (*n* = 10), and EcN (*n* = 10). Statistical significance among groups was evaluated by one-way ANOVA followed by the Tukey’s test. Bars with different letters are significantly different (*p* < 0.05). Non-colitic: untreated healthy group (*n* = 10); DSS-colitic: untreated DSS-induced colitic group (*n* = 10) and EcN: DSS-induced colitic group treated with probiotic (*n* = 10).

Male C57BL/6J mice (7–9 weeks old; approximately 20 g) obtained from Janvier (Saint-Berthevin Cedex, France) were randomly assigned to three different groups of 10 animals each. The non-colitic and the DSS-colitic groups received PBS solution (200 μL), whereas the EcN group was daily treated with the probiotic EcN at the concentration of 5 × 10^8^ CFU, by using an oesophageal catheter, for 26 days. Two weeks after the onset of the study, colitis was induced in the DSS-colitic group and in the EcN group by adding 3% DSS (36–50 kDa, MP Biomedicals, Ontario, CA, United States) to drinking water for 6 days; subsequently, DSS was removed (Mahler et al., 1998; **Figure 1A**). All the mice were killed 26 days after the onset of the study with an overdose of halothane.

Animal body weight, the presence of gross blood in the feces, and the stool consistency were evaluated daily for each mouse by a blind observer. A score was assigned to each one of these parameters according to the criteria previously proposed. These scores were used to calculate an average daily of the DAI (Camuesco et al., 2004). Once the animals were killed, the colon was removed aseptically, opened longitudinally, and all the contents collected; then it was weighed, and its length was measured under a constant load (2 g; Sanchez de Medina et al., 1996). Representative specimens (0.5 cm length containing all wall layers) were taken from the distal inflamed region and fixed in 4% buffered formaldehyde for histological analysis. The remaining colonic tissue and the fecal content were immediately stored at –80°C until analysis.

### Histological Studies

The formalin-fixed colonic specimens were paraffin-embedded, sectioned (5 μm) at different levels and stained with alcian blue and hematoxylin and eosin. The histological damage was assessed by a blind observer according to a previous reported histologic scoring system, which takes into account the presence of ulceration, infiltration, edema, and the condition of the crypts (Camuesco et al., 2012).

### DNA Extraction and 454/Roche Pyrosequence Analysis

A representative sample of each group (non-colitic, *n* = 3; DSS-colitic, *n* = 4; and EcN, *n* = 3) was chosen to carry out the microbiota analysis by pyrosequencing. Phenol:chloroform (Sambrook and Russell, 2006) was used to isolate DNA from fecal samples. Total DNA isolated from the stool samples was amplified by PCR to compare the effect of different storage and purification methods on the recovery of 16S rRNA gene sequence. Primers targeting regions flanking the variable regions 1–3 of the bacterial 16S rRNA gene (V1-3) were used. The gel was purified and analyzed using the Roche 454 GS Junior System (Roche Diagnostics, Branford, CT, United States). The amplification of a 600-bp sequence in the variable region V1–V3 of the 16S rRNA gene was performed using bar-coded primers. The PCR was performed in a total volume of 15 μL containing the universal 27F and Bif16S-F forward primers (10 μmol L^-1^) at a 9:1 ratio, and the bar-coded universal reverse primer 534R (10 μmol L^-1^), dNTP mix (10 mmol L^-1^), Fast Start 10 × buffer with 18 mmol L^-1^ of MgCl_2_, Fast Start HiFi polymerase (5 U in 1 mL), and 2 μL of genomic DNA. The dNTP mix, Fast Start 10 × buffer with MgCl_2_, and Fast Start HiFi polymerase were included in a FastStart High Fidelity PCR System, dNTP Pack (Roche Applied Science, Penzberg, Germany). The PCR conditions were as follows: 95°C for 2 min, 30 cycles of 95°C for 20 s, 56°C for 30 s, and 72°C for 5 min, and a final step at 4°C. After the PCR, amplicons were further purified using AMPure XP beads (Beckman Coulter, Inc., Indianapolis, IN, United States) to remove smaller fragments. The DNA concentration and quality were measured using a Quant-iT^TM^ PicoGreen^®^ dsDNA Assay Kit (Thermo Fisher Scientific, Waltham, MA, United States). Finally, the PCR amplicons were combined in equimolar ratios to create a DNA pool (10^9^ DNA molecules) that was used for emPCR and pyrosequencing according to the manufacturer’s instructions (Roche 454 GS Junior System; Roche Diagnostics, Branford, CT, United States). Once the sequencing was completed, all reads were scored for quality, and any poor quality and short reads were removed.

### Taxonomic Analysis

Sequences were selected to estimate the total bacterial diversity of the DNA samples in a comparable manner and were trimmed to remove barcodes, primers, chimeras, plasmids, mitochondrial DNA, and any non-16S bacterial reads and sequences < 150 bp. The MG-RAST (Meyer et al., 2008) with the RDP was used for analyses of all sequences. The pipeline takes in bar-coded sequence reads, separates them into individual communities by bar code, and utilizes a suite of external programs to make taxonomic assignments RDP database (Wang et al., 2007) and estimates phylogenetic diversity, with a minimum e-value of 1e-5, a minimum identity of 60%, and a minimum alignment length of 15 measured in base pairs for RNA databases. Each value obtained indicated the percentage of relative frequency of reads with predicted proteins and ribosomal RNA genes annotated to the indicated taxonomic level. The output file was further analyzed using SPSS Statistics 17.0 Software Package (SPSS Inc.) and Statistical Analysis of Metagenomic Profiles (STAMP) software package version 2.1.3 (Parks et al., 2014).

### Availability of Supporting Data

Metagenomic data sets for all samples are available on the MG-RAST web server^1^, being sample IDs as follows: non-colitic, 4548801.3, 4548800.3, 4548793.3; DSS-colitic, 4548805.3, 4548804.3, 4548803.3, 4548802.3; and EcN, 4548806.3, 4548807.3, 4548784.3.

### Analysis of Gene Expression in Mouse Colonic Samples by RT-qPCR

Total RNA from colonic samples was isolated using the RNeasy^®^ Mini Kit (Qiagen, Hilden, Germany) following the manufacturer’s protocol. All RNA samples were quantified with the Thermo Scientific NanoDropTM 2000 Spectrophotometer (Thermo Fisher Scientific, Waltham, MA, United States) and 2 μg of RNA was reverse transcribed using oligo (dT) primers (Promega, Southampton, United Kingdom).

Real-time quantitative PCR amplification and detection was performed on optical-grade 48-well plates in an EcoTM Real-Time PCR System (Illumina, CA, United States). Each reaction contained 5 μL of KAPA SYBR^®^ FAST qPCR Master Mix (Kappa Biosystems, Inc., Wilmington, MA, United States), each amplification primer at a concentration of 10 μM, 20 ng of cDNA from the RT reaction, and PCR-grade water up to a final volume of 10 μL.

The thermal cycling program was as follows: an initial denaturation step of 10 min at 95°C, followed by 40 cycles of 15 s at 95°C and 1 min at annealing temperature (55–62°C). Fluorescence was measured at the end of the annealing period of each cycle to monitor the progress of amplification, and dissociation curves were added to confirm the specificity of the amplification signal in each case. The expression of the housekeeping gene, GAPDH, was measured to normalize mRNA expression of target genes. For each sample, both the housekeeping and the target genes were amplified in triplicate and the mean value was used for further calculations. The mRNA relative quantitation was done using the ΔΔCt method. **Table 1** shows the primers used.

**Table 1:** Primer sequences used in real-time qPCR assays.

Gene	Sequence (5′-3′)	Annealing temperature (°C)
*IL-1β*	FW:TGATGAGAATGACCTGTTCT	55
	RV:CTTCTTCAAAGATGAAGGAA	
*IL-12*	FW:CCTGGGTGAGCCGACAGAAGC	60
	RV:CCACTCCTGGAACCTAAGCAC	
*TGF-β*	FW:GCTAATGGTGGACCGCAACAAC	60
	RV:CACTGCTTCCCGAATGTCTGAC	
*ICAM-1*	FW:GAGGAGGTGAATGTATAAGTTATG	60
	RV:GGATGTGGAGGAGCAGAG	
*MUC-2*	FW:GATAGGTGGCAGACAGGAGA	60
	RV:GCTGACGAGTGGTTGGTGAATG	
*MUC-3*	FW:CGTGGTCAACTGCGAGAATGG	62
	RV:CGGCTCTATCTCTACGCTCTCC	
*ZO-1*	FW:GGGGCCTACACTGATCAAGA	56
	RV:TGGAGATGAGGCTTCTGCTT	
*OCLN*	FW:ACGGACCCTGACCACTATGA	56
	RV:TCAGCAGCAGCCATGTACTC	
*GAPDH*	FW:CATTGACCTCAACTACATGG	60
	RV:GTGAGCTTCCCGTTCAGC	
*miR-143*	UGAGAUGAAGCACUGUAGCUC	55
*miR-150*	UCUCCCAACCCUUGUACCAGUG	55
*miR-155*	UUAAUGCUAAUUGUGAUAGGGGU	55
*miR-223*	UGUCAGUUUGUCAAAUACCCCA	55
*miR-375*	UUUGUUCGUUCGGCUCGCGUGA	55
*SNORD95*	TATTGCACTTGTCCCGGCCTGT	55

The miRNA from colonic samples was isolated after homogenizing the tissue in QIAzol^TM^ (Qiagen, Hilden, Germany) using a Precellys^®^24 homogenizer (Bertin Technologies, Montigny-le-Bretonneux, France). Small RNA (<200 nucleotides) fractions were isolated separately using the miRNeasy mini Kit (Qiagen, Hilden, Germany) according to the Supplementary Protocol provided by the manufacturer.

All miRNA samples were quantified with the Thermo Scientific NanoDropTM 2000 Spectrophotometer (Thermo Fisher Scientific, Waltham, MA, United States) and 500 ng of miRNA was reverse transcribed using the miScript II RT kit from Qiagen (Qiagen, Hilden, Germany).

Quantitative real-time PCR amplification and detection was performed on optical-grade 48-well plates in an EcoTM Real-Time PCR System (Illumina, CA, United States). Each reaction contained 5 μL QuantiTect SYBR Green PCR Master Mix (Qiagen, Hilden, Germany), 1 μL miScript Universal Primer, 1 μL miScript Primer Assay, 2 ng of cDNA from the RT reaction, and PCR-grade water up to a final volume of 10 μL.

The thermal cycling program was as follows: an initial activation step of 15 min at 95°C, followed by 40 cycles with three-step cycling: 15 s at 94°C for denaturation, the annealing step at 55°C for 30 s and 30 s at 70°C for the extension step. Fluorescence was measured at the extension period of each cycle to monitor the progress of amplification, and dissociation curves were added to confirm the specificity of the amplification signal in each case. The expression of the housekeeping gene, SNORD95, was measured to normalize miRNA expression. For each sample, both the housekeeping and target genes were amplified in triplicate and the mean was used for further calculations. The miRNA relative quantitation was done using the ΔΔCt method. **Table 1** shows the primers used.

### Statistics

All statistical analyses were carried out with the GraphPad Prism version 6.0 (GraphPad Software Inc., La Jolla, CA, United States), with statistical significance set at *p* < 0.05. DAI values and weight evolution in DSS-colitic mice were analyzed using one-way analysis of variance (ANOVA) followed by Dunnett’s test. Parametric comparisons were done using one-way ANOVA followed by Tukey’s test. All variables are described as mean ± SEM.

## Results

**FIGURE 2:**
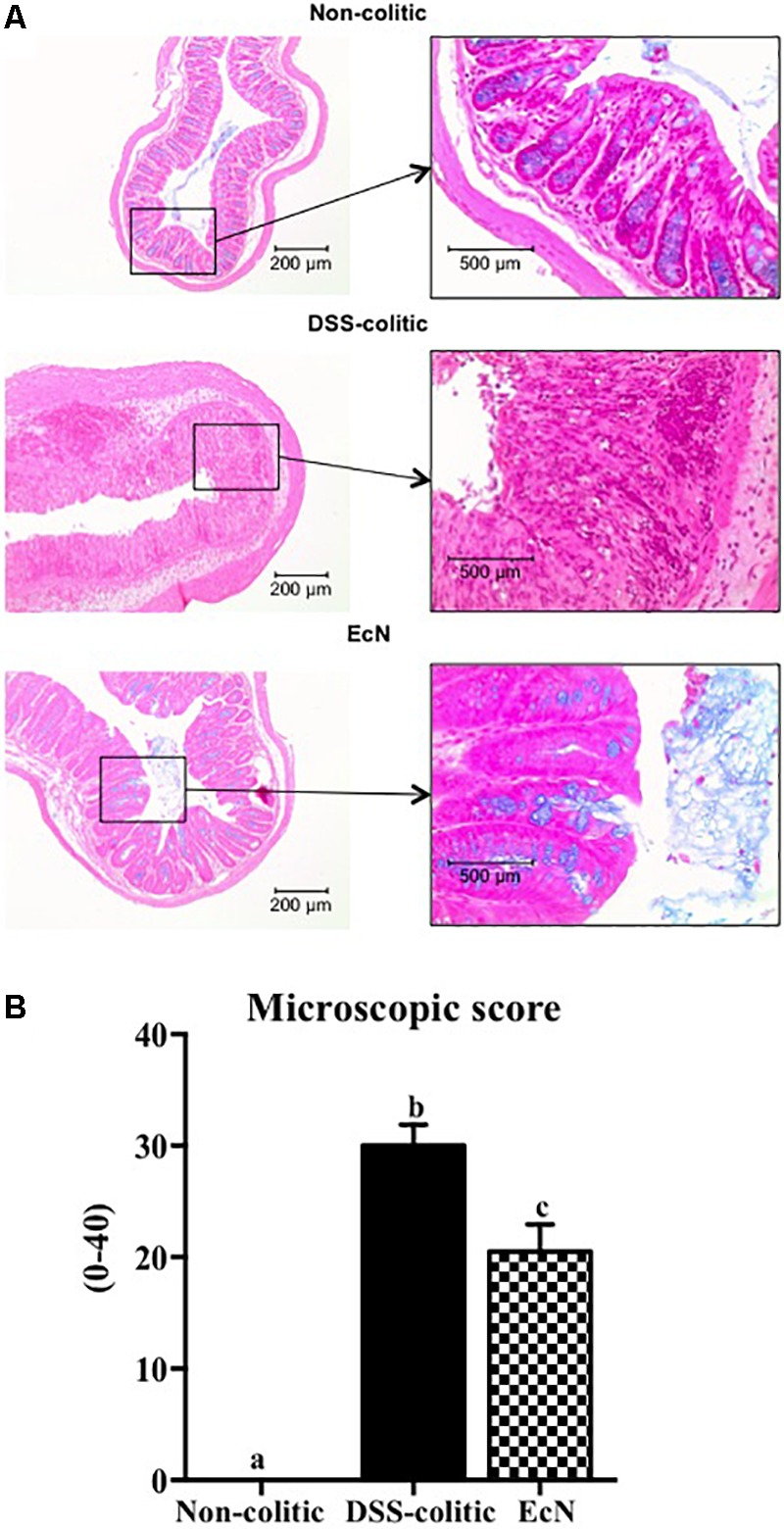
*Escherichia coli* Nissle 1917 (EcN) treatment promotes recovery of DSS-induced intestinal injury and inflammation in mice. **(A)** Histological images of colonic tissue stained with hematoxylin and eosin showing the effect of EcN on DSS-induced colitis. Representative images of each experimental group are shown: non-colitic, DSS-colitic, and EcN. In non-colitic mice, the images show the normal appearance of the intact mucosa containing the crypts with goblet cells full of mucin. In DSS-colitic group, the images show changes in the mucosa with areas of ulceration on the epithelial layer, in addition to a lower number of goblet cells which were depleted in mucin and an intense inflammatory cell infiltrate. In EcN group, an improvement of the colonic histology is found, with a reduced area of ulceration, mostly in process of recovery, presence of goblet cells replenished with their mucin content and reduced inflammatory cell infiltrate. **(B)** Histological scores calculated after microscopic analyses of longitudinal colon sections. Results are expressed as mean ± SEM. Statistical significance among groups was evaluated by one-way ANOVA followed by the Tukey’s test. Different letters denote significant differences between groups (*p* < 0.05). Non-colitic: untreated healthy group (*n* = 10); DSS-colitic: untreated DSS-induced colitic group (*n* = 10) and EcN: DSS-induced colitic group treated with probiotic (*n* = 10).

In comparison with the non-treated group (non-colitic and DSS-colitic), the administration of EcN to mice belonging to the EcN group did not show any sign of toxicity, which was evaluated by body weight increase, food intake, and general appearance of the animals. The addition of 3% (w/v) of DSS to drinking water for 6 days resulted in a progressive increase in DAI values in the DSS-colitic group, due to body weight loss and excretion of diarrheic/bleeding feces (**Figure 1A**). However, the oral probiotic treatment attenuated the impact of the DSS damage and boosted the recovery of the colitic mice (**Figure 1B**). These effects were evidenced by a reduction in body weight loss and by a lower incidence of diarrheic/bloody feces, which resulted in significantly lower DAI values throughout the experiment in the EcN group when compared to untreated colitic group (0.8 ± 0.015 for EcN group vs. 1.72 ± 0.02 for DSS-colitic group; **Figure 1A**). The macroscopic evaluation of the colonic segments confirmed the beneficial effects found in probiotic-treated colitic mice (EcN group), since they showed a significant reduction in colonic weight/length ratio compared to the corresponding DSS-colitic group (-23.7%, *p* < 0.05; **Figure 1C**). Of note, it has been suggested that this ratio is directly correlated to the severity of the colonic damage in this experimental model of colitis (Arafa et al., 2009). Microscopically, the colonic inflammation in the DSS-colitic group was characterized by an intense ulceration affecting more than 75% of the surface and a complete mucin depletion from goblet cells. In addition, severe leukocyte infiltration and edema were frequently found in DSS-colitic group. The damage was considered as very severe in most of the sections, assigning a microscopic score (mean ± SEM) of 30.0 ± 1.9 to the DSS-colitic group. The administration of EcN improved the epithelial regeneration, showing a reduction in the colonic ulcerated area, which was below 50% for all samples, and preserved the goblet cells and their mucin content. Moreover, the probiotic treatment decreased the inflammatory cell infiltration and the edema in the mucosa, obtaining a significant reduction in the microscopic score of the EcN group (20.0 ± 2.5; *p* < 0.05) compared to the DSS-colitic group (**Figure 2**).

**FIGURE 3:**
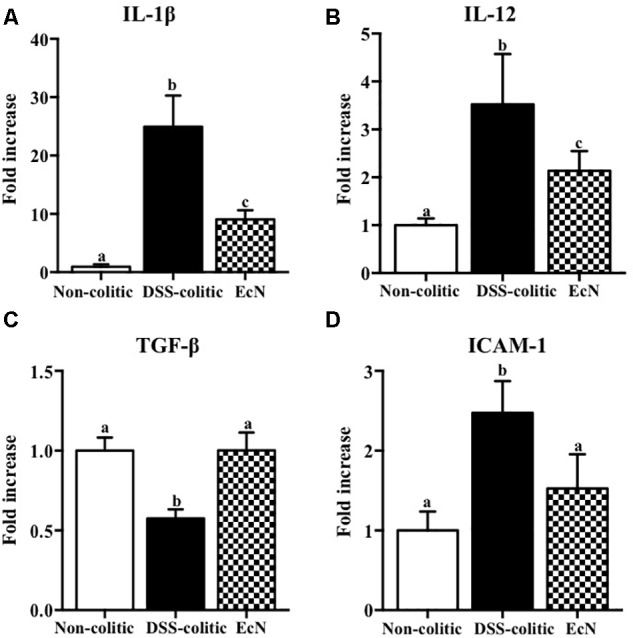
Biochemical evaluation of the effects of *Escherichia coli* Nissle 1917 (EcN); mRNA expression of cytokines **(A)** IL-1β, **(B)** IL-12, **(C)** TGF-β, and **(D)** ICAM-1 was quantified by real-time PCR, and fold changes are expressed as means ± SEM. Non-colitic group (*n* = 10), DSS-colitic group (*n* = 10), and EcN group (*n* = 10). Statistical analysis was performed with one-way ANOVA followed by Tukey’s test. Bars with different letters are significantly different (*p* < 0.05). Non-colitic: untreated healthy group (*n* = 10); DSS-colitic: untreated DSS-induced colitic group (*n* = 10) and EcN: DSS-induced colitic group treated with probiotic (*n* = 10).

**FIGURE 4:**
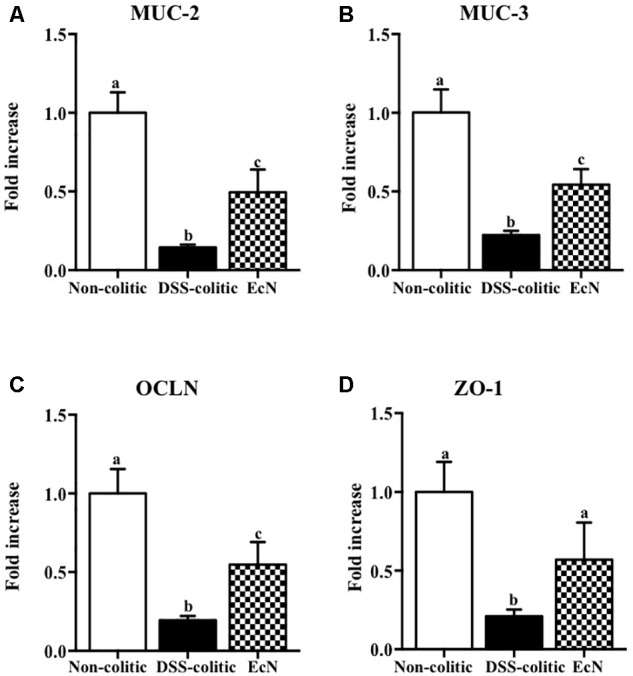
Biochemical evaluation of the effects of *Escherichia coli* Nissle 1917 (EcN); mRNA expression of epithelial integrity proteins, **(A)** MUC-2, **(B)** MUC-3, **(C)** occludin (OCLN), and **(D)** zonula occludens-1 (ZO-1), was quantified by real-time PCR, and fold changes are expressed as means ± SEM. Non-colitic group (*n* = 10), DSS-colitic group (*n* = 10), and EcN group (*n* = 10). Statistical analysis was performed with one-way ANOVA followed by Tukey’s test. Bars with different letters are significantly different (*p* < 0.05). Non-colitic: untreated healthy group (*n* = 10); DSS-colitic: untreated DSS-induced colitic group (*n* = 10) and EcN: DSS-induced colitic group treated with probiotic (*n* = 10).

The biochemical analysis of the colonic segments corroborated the intestinal anti-inflammatory effect of this probiotic, thus revealing its positive impact on the altered colonic immune response induced by DSS intake. In this sense, the probiotic significantly reduced the up-regulated expression of the pro-inflammatory cytokines IL-1β and IL-12 found in EcN group (-63.55% and -39.29%, *p* < 0.05, respectively; **Figure 3**). The colonic inflammatory process was associated with a 50% decrease of the TGF-β expression in EcN group compared to the non-colitic group (*p* < 0.05). The TGF-β is a cytokine that displays both anti-inflammatory and pro-inflammatory properties, depending on the local cytokine milieu. However, the probiotic treatment restored this cytokine expression to values similar to non-colitic mice (**Figure 3**). EcN was also able to significantly reduce by 38.58% the increased expression of the adhesion molecule ICAM-1 found in DSS-colitic mice (*p* < 0.05; **Figure 3**). As it has been previously described in this experimental model of colitis (Garrido-Mesa et al., 2011, 2015), the colonic inflammation was associated with a reduced expression of different proteins involved in the epithelial integrity and barrier function of the colonic mucosa, like the mucins MUC-3 and MUC-2, as well as OCLN and ZO-1. The administration of this probiotic to colitic mice showed a significant amelioration in the expression of all these markers (**Figure 4**).

**FIGURE 5:**
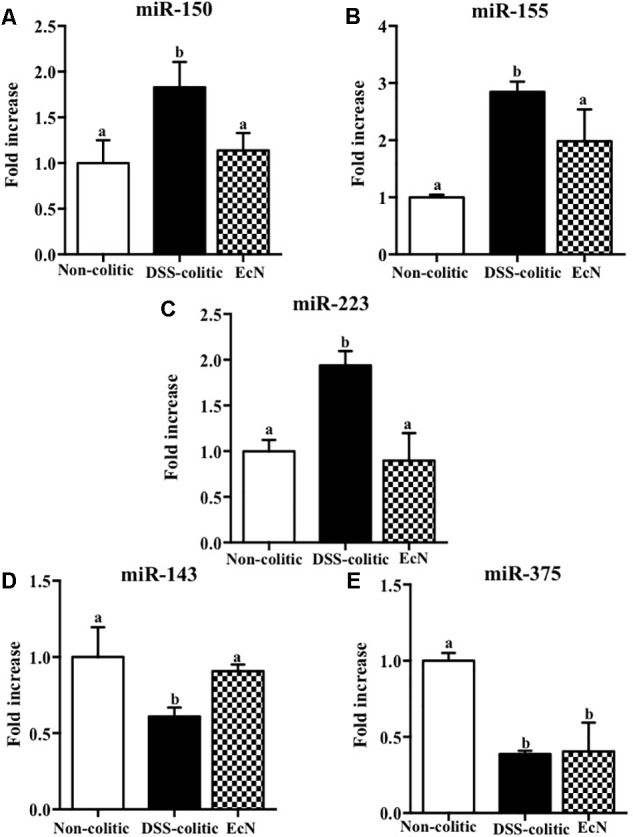
Biochemical evaluation of the effects of *Escherichia coli* Nissle 1917 (EcN); the expression of **(A)** miR-150, **(B)** miR-155, **(C)** miR-223, **(D)** miR-143, and **(E)** miR-375 was quantified by real-time PCR. Fold changes are expressed as means ± SEM. Non-colitic group (*n* = 10), DSS-colitic group (*n* = 10), and EcN group (*n* = 10). Statistical analysis was performed with one-way ANOVA followed by Tukey’s test. Bars with different letters are significantly different (*p* < 0.05). Non-colitic: untreated healthy group (*n* = 10); DSS-colitic: untreated DSS-induced colitic group (*n* = 10) and EcN: DSS-induced colitic group treated with probiotic (*n* = 10).

The results obtained in this experimental model of DSS-induced colitis confirmed that the intestinal inflammatory process was associated with noticeable molecular changes in gene expression. Also, these results support that the restoration of the altered immune response is involved in the preventive beneficial effects exerted by the probiotic assayed in experimental colitis. Then, we explored if the expression of miRNAs, which have been reported to play an important role in many biological processes, such as signal transduction, cellular proliferation, differentiation, apoptosis, and immune response (O’Connell et al., 2012), could also be associated with the response to probiotic treatment. We conducted a customized array of 80 different miRNAs chosen based on an inflammatory panel found in the colonic tissue of mice with DSS-induced colitis. In the present study, we selected five of the eleven changed miRNA, specifically miR-143, miR-150, miR-155, miR-223, and miR-375, and questioned whether their expression was altered following treatment of DSS-colitis mice with *E. coli* Nissle. The expressions of these particular miRNAs were evaluated by qPCR. Results showed that three of them, miR-150, miR-155, and miR-223, were significantly upregulated (around twofold increase vs. healthy control, *p* < 0.05) whereas the other two, miR-143 and miR-375, were significantly downregulated in colitic mice compared to non-colitic group (around twofold decrease, *p* < 0.05). The treatment with the probiotic showed an amelioration of the altered expression in some of the assessed miRNAs. Thus, EcN was able to significantly reduce the upregulated expressions of miR-155 and miR-223 and downregulate miR-150 expression in comparison with DSS-colitic mice (-37.7%, *p* < 0.05; **Figure 5**). Moreover, the reduced expression of miR-143 in colitic mice was also restored by the probiotic treatment (0.91 ± 0.06 in EcN group vs. 1.00 ± 0.39 in DSS-colitic group, *p* > 0.05; **Figure 5**).

**FIGURE 6:**
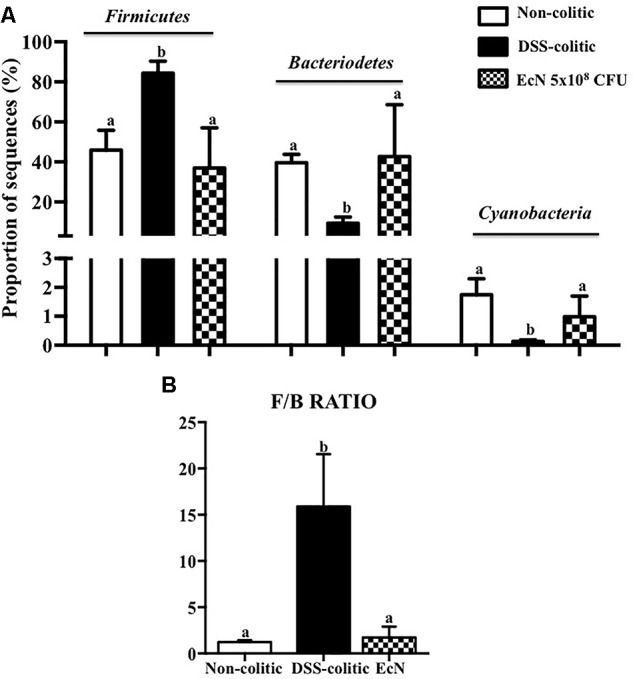
Comparison of microbiota composition between non-colitic group (*n* = 3), DSS-colitic group (*n* = 4), and *Escherichia coli* Nissle 1917 (EcN) group (*n* = 3). **(A)** Phylum breakdown of the most abundant bacterial communities in the different groups. Results were expressed as the mean ± SEM of the proportion of sequences and compared by one-way ANOVA followed by Tukey’s test. **(B)** The *Firmicutes*/*Bacteriodetes* ratio (F/B ratio) was calculated as a biomarker of gut dysbiosis. Statistical analysis was performed with one-way ANOVA followed by Tukey’s test. Bars with different letters are significantly different (*p* < 0.05).

**FIGURE 7:**
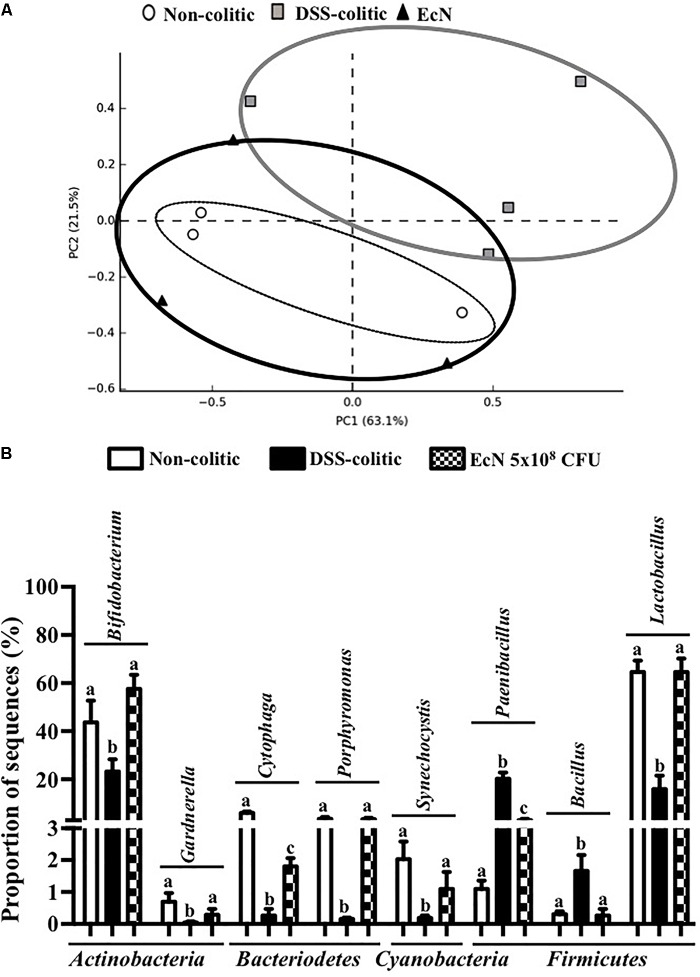
**(A)** Principal component analysis plot based on Bray–Curtis distances, calculated on the metagenomic table of fecal samples of the different groups [non-colitic (*n* = 3), DSS-colitic (*n* = 4), and *Escherichia coli* Nissle 1917 (EcN; *n* = 3)]. **(B)** Mean percentage of sequences of the most important genera in DSS-induced colitis model. Results were shown as the means ± SEM and compared by one-way ANOVA followed by Tukey’s test. Bars with different letters are significantly different (*p* < 0.05).

Subsequently, 16S ribosomal DNA sequencing and bioinformatics alignment comparison against RDP database were performed to study the modifications in the intestinal microbiota composition in colitic mice after a DSS insult. The most abundant phylum in the gut microbiota in the analyzed samples was *Firmicutes*. However, significant differences in microbial content were found among the different groups at the phylum level (**Figure 6A**). The *Firmicutes* phylum was significantly increased in the DSS-colitic group compared to non-colitic mice (+86%, *p* < 0.05). The treatment with EcN was able to attenuate this increase (*p* > 0.05 vs. healthy control; **Figure 6A**). Significant reductions in *Cyanobacteria* and *Bacteriodetes* phyla were found in the DSS-colitic group compared with non-colitic mice (-76.6 and -92.6%, respectively, *p* < 0.05). The probiotic treatment used in the present study significantly increased *Cyanobacteria*, and increased the proportion of sequences of *Bacteriodetes* up to a value similar to that found in non-colitic mice (*p* > 0.05; **Figure 6A**). Therefore, the F/B ratio was significantly increased (13-fold) in the DSS-colitic group compared to the non-colitic mice. This ratio, which has been used as a biomarker for different pathological conditions including intestinal inflammation (Sanz and Moya-Perez, 2014), was partially restored by EcN treatment (**Figure 6B**). Based on the ordination plot of the distance matrix generated using Bray–Curtis complementary algorithm, a clear demarcation between bacterial assemblages from healthy and DSS-colitic groups along principal coordinate axis 1 (PC1) of the principal coordinate analysis (PCA) plot was found. The PCA plot showed that the communities found in the probiotic-treated colitic mice (EcN group) clearly differed from those in the DSS-colitic group, but were quite similar to those found in the non-colitic mice (*p* > 0.05; **Figure 7A**).

Additionally, significant changes in the proportion of sequences of several bacterial genera were also found in DSS-colitic mice compared to non-colitic group, including *Bifidobacterium*, *Bacillus*, *Cytophaga*, *Gardnerella*, *Lactobacillus*, *Paenibacillus*, *Porphyromonas*, and *Synechocystis* (**Figure 7B**). The probiotic used in the present study was able to restore the composition in all modified bacterial genera (**Figure 7A**).

**FIGURE 8:**
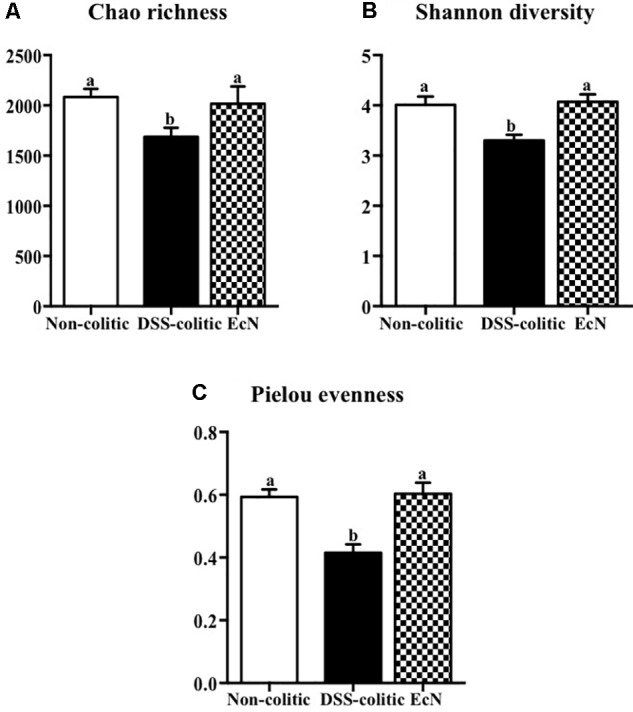
Estimation of the phylogenetic diversity of the gut microbiota found in the different experimental groups; non-colitic (*n* = 3), DSS-colitic (*n* = 4), and *Escherichia coli* Nissle 1917 (EcN; *n* = 3) using the **(A)** Chao richness, **(B)** Shannon diversity, and **(C)** Pielou evenness. The values are means, and error bars with different letters are significantly different (*p* < 0.05). Statistical analysis was performed with one-way ANOVA followed by Tukey’s test. Non-colitic: untreated healthy group (*n* = 3); DSS-colitic: untreated DSS-induced colitic group (*n* = 4) and EcN: DSS-induced colitic group treated with probiotic (*n* = 3).

Finally, several ecological features of the gut bacterial communities were evaluated in the three experimental groups. A variety of indices at the operational taxonomic unit (OTU) level were determined. In particular, the compositions of bacterial communities were characterized by calculating three major ecological parameters, including Chao richness (an estimate of a total community), Pielou evenness (showing how evenly the individuals are distributed in the community over different OTUs), and Shannon diversity (the combined parameter of richness and evenness). Microbial richness, evenness, and diversity were significantly decreased in the DSS-colitic group compared to the non-colitic group. Importantly, the administration of EcN to colitic mice was able to restore all these ecological parameters to normal values (**Figure 8**).

## Discussion

Inflammatory bowel disease is characterized by an exacerbated immune response that leads to chronic inflammation, associated with drastic changes in gene and protein expression. This could be partially ascribed to miRNAs, as they participate in many of the signaling pathways involved. An imbalance in the intestinal microbiota composition has been reported to contribute to the pathogenesis of IBD (Kostic et al., 2014), and many studies focus on the development of new strategies to restore it. They include the administration of probiotics that exert beneficial effects in human IBD and in experimental models of colitis (Grabig et al., 2006; Garrido-Mesa et al., 2011). The present study corroborates the intestinal anti-inflammatory effects of the probiotic, EcN, which improved both macroscopic and biochemical parameters in the DSS model of mouse colitis.

The administration of EcN enhanced the altered immune response in colitic mice, thus confirming their intestinal immuno-modulatory properties (Stephani et al., 2011). Therefore, probiotic treatment restored the normal expression of the pro-inflammatory cytokines IL-1β, TGF-β, and IL-12. These cytokines are considered important inflammatory mediators of innate and/or adaptive immunity, driving intestinal inflammation (Korzenik and Podolsky, 2006). Similarly, other inflammatory mediators, like chemokines or adhesion molecules, are overexpressed in colitic mice Interestingly, EcN reduced ICAM-1 expression, which could impair neutrophils and macrophages access to intestinal inflamed areas. This effect has been already reported, thus demonstrating a protective effect against the acute inflammatory process (Souza et al., 2016). Additionally, another initial event in intestinal inflammation is the impairment of the epithelial barrier function (Mankertz and Schulzke, 2007). Indeed, a defect in the mucus layer that covers the epithelium and protects its integrity has been described in human IBD (Gersemann et al., 2009) and in experimental models of rodent colitis (McGuckin et al., 2009). In the present study, DSS-induced colitis was also associated with a reduction in the expression of mucins, MUC-2 and MUC-3, and tight junction proteins, OCLN and ZO-1, which participate in maintaining epithelial integrity (Kim and Ho, 2010). The probiotic used in the present study significantly upregulated the expression of the mucins as well as OCLN and ZO-1. This effect could preserve the mucus-secreting layer and facilitate the restoration of the epithelial barrier function and integrity, thus improving intestinal permeability and promoting colonic recovery, in consistency with previously described for probiotics (Persborn and Soderholm, 2013). Regarding the study of the role of miRNAs in the etiopathogenesis of intestinal inflammation and the effect of EcN, we focused on five miRNAs, based on previous studies in DSS-induced colitis (Rodriguez-Nogales et al., 2017). Both miR-150 and miR-155 have been involved in the regulation of the immune response by controlling the development and function of innate immune cells. The expression of these miRNAs is modulated by pro-/anti-inflammatory signals, including cytokines, which in turn participate in the release of other cytokines and chemokines (O’Connell et al., 2010, 2012). Accordingly, the expression of both miR-150 and miR-155 has been reported to be up-regulated in colonic epithelial cells in UC patients and in colitic mice (Bian et al., 2011; Singh et al., 2014; Lu et al., 2017). This upregulation has been confirmed in the present study. However, EcN decreased the colonic expression of both miRNAs, whereas it increased miR-150 expression, which could facilitate colonic epithelial cell apoptosis via reduction of c-Myb (Bian et al., 2011). In fact, the decreased expression of c-Myb found in DSS-induced colitis may promote epithelial disruption and increase permeability of the gut. Therefore, EcN could protect epithelial barrier integrity and restore the altered immune response. The expression of miR-155 has been reported to be upregulated in B and T lymphocytes, as well as in macrophages, following antigen activation (O’Connell et al., 2010). Moreover, miR-155^-/-^ mice are protected from DSS-induced colitis, likely through the reduction in the upregulated Th1/Th17 responses that characterize DSS colitis (Singh et al., 2014). Accordingly, the ability showed by EcN to reduce the colonic expression of miR-155 in colitic mice could account for its immuno-modulatory properties, also evidenced by the downregulation of the pro-inflammatory cytokines IL-1β and IL-12, in conjunction with the upregulation of the anti-inflammatory cytokine TGF-β.

Similarly, an upregulation of miR-223 occurs in inflammatory conditions; this miRNA is involved in the inflammosome complex modulation and in IL-1β production (Haneklaus et al., 2012). The probiotic administration in our study was able to significantly reduce the expression of this miRNA and IL-1β in colitic mice. Additionally, miR-143 seems to play an important role in maintaining the normal colonic biology; thus, when it downregulates the pro-inflammatory signals of the innate immune system appear (Starczynowski et al., 2010). Actually, the colonic expression of miR-143 was significantly downregulated in the DSS-colitic mice. However, EcN was able to restore its colonic expression, thus preserving colonic function and an adequate innate immune response. Finally, miR-375 is considered a multifunctional miRNA, involved in different processes including pancreatic islet development, glucose homeostasis, or cell differentiation and carcinogenesis (Xu et al., 2011); miR-375 displays different expression profiles depending on the disease considered (Zhao et al., 2012). In experimental colitis, a downregulation of miR-375 expression in IL-10^-/-^ mice once the signs of the colonic inflammation were evident was reported (Schaefer et al., 2011). Similarly, in our study, the expression of miR-375 was significantly reduced in colitic mice in comparison with non-colitic ones. However, the probiotic used by us failed to restore the basal levels of miR-375. It is noteworthy that only few studies have evaluated the capacity of probiotics to modulate miRNAs expression, and most of them have been performed *in vitro* (Giahi et al., 2012). Therefore, these results could show that the administration of the probiotic EcN to colitic mice improve the altered immune response, maybe by modifying miRNA expression in colonic tissue. Recently, host miRNAs capable to specifically modulate gut microbiome have been identified (DuPont and DuPont, 2011). In addition, it has been suggested that probiotic administration reverses dysbiosis and restores tolerance toward gut microbiota in these intestinal conditions (DuPont and DuPont, 2011). Therefore, we suggest that EcN, which can modulate different miRNAs’ expression, could also restore the gut microbiota. This potential ability of this probiotic has not been previously reported. Therefore, the present study aims to characterize the different changes occurring in gut microbiota by the action of this probiotic. First, three ecological parameters were calculated, Chao richness, Pielou evenness, and Shannon diversity, which were significantly reduced in the DSS-colitic group when compared to the non-colitic group, as previously reported in IBD patients (Frank et al., 2007; Walker et al., 2011). Regarding the treatment, EcN restored the three ecological parameters to healthy values. Moreover, the F/B ratio increased in DSS-colitic mice compared to non-colitic mice; this change in the ratio is associated with a significant expansion of *Firmicutes* and a reduction of *Bacteriodetes*. EcN significantly reduced F/B ratio in EcN group compared to DSS-colitic group. These results seem inconsistent with some metagenomic studies conducted in experimental models of intestinal inflammation and in IBD patients which have previously reported a reduction in the bacterial phylum *Firmicutes* (Frank et al., 2007; Atarashi and Honda, 2011). However, other studies have described increased levels of different subgroups of *Firmicutes*, including those that include bacteria like *Clostridium difficile*, which seems to play a key role in the pathogenesis of these conditions (Kim and Milner, 2007; Kim and Ho, 2010). Moreover, the ability showed by EcN to produce an expansion of the *Bacteriodetes* phylum in colitic mice can also contribute to its beneficial effects, since *Bacteroidetes* are responsible for keeping the homeostasis in the gastrointestinal tract (Kim and Milner, 2007). The bacterial populations that produce SCFAs, like butyrate, propionate, and acetate, also contribute to normal gut homeostasis. Although certain human-derived butyrate-producing strain could aggravated colitis in DSS-treated mice exerting no detrimental effect in healthy mice, the SCFAs, and mainly butyrate, regulate epithelial cell differentiation, barrier function, and stimulation of regulatory T cells, being able to ameliorate mucosal inflammation in IBD conditions (Hartog et al., 2015; Zhang et al., 2016). Interestingly, butyrate main producer bacteria from the genera *Bifidobacterium* and *Lactobacillus* (Manichanh et al., 2006) were reduced in DSS-colitic mice, while both genera increased by EcN treatment. Accordingly, the positive impact exerted by EcN on these bacterial populations may also contribute to the beneficial intestinal anti-inflammatory effects of this probiotic.

## Conclusion

The present study demonstrates that the prophylactic administration of the probiotic EcN displays an intestinal anti-inflammatory effect in the experimental model of DSS-induced acute colitis in mice, in consistency with previous studies (Schultz et al., 2004; Garrido-Mesa et al., 2011). The mechanisms involved could be associated with the immuno-modulatory capacity of EcN. Regarding the latter, this probiotic treatment shows a positive impact on the innate immune response, preserving intestinal barrier integrity and decreasing pro-inflammatory cytokines production. Concerning the adaptive immune response, EcN modulates the expression of Th1-, Th17- and Treg-related cytokines. These actions could be achieved at a post-transcriptional level by modifying the expression of some miRNAs altered in inflammatory conditions. The restoration of miRNAs expression by EcN pre-treatment was associated with its ability to ameliorate the gut dysfunction by improving microbial homeostasis, compromised after DSS administration. This is the first study that describes the mechanism of action of EcN through miRNAs this mechanism in the DSS model of experimental colitis, since many studies have demonstrated the ability of probiotics to improve dysbiosis, but just a few studies have linked probiotics to their capacity to modulate miRNAs expression and different mediators of the immune response involved in gut inflammation. This could be of great interest to understand the mechanism of action of this probiotic in the treatment of IBD.

## Data Availability

Data can be requested from the corresponding author.

## Author Contributions

AR-N, FA, JG-M, MU, TV, and MR-C performed the experiments and contributed to the acquisition and analysis of data. AR-N, NC, JF-C, and FG contributed to the taxonomic analysis and data interpretation. AR-N, FA, MU, MR-C, and JG designed the experiments and wrote the manuscript.

## Conflict of Interest Statement

The authors declare that the research was conducted in the absence of any commercial or financial relationships that could be construed as a potential conflict of interest.

## Footnotes

1 http://metagenomics.anl.gov/

## Abbreviations

CD: Crohn’s disease
CFU: colony-forming units
DAI: disease activity index
DSS: dextran sodium sulfate
EcN: *Escherichia coli* Nissle 1917
emPCR: emulsion-based clonal amplification
F/B: *Firmicutes*/*Bacteroidetes*
GAPDH: glyceraldehyde-3-phosphate dehydrogenase
IBD: inflammatory bowel disease
MAPKs: mitogen-activated protein kinases
MG-RAST: metagenomics analysis server
miRNAs/mi-R: micro-RNAs
NF-κB: nuclear factor-κB
OCLN: occludin
PCR: polymerase chain reaction
qPCR: real-time quantitative PCR
RDP: Ribosomal Database Project
RT: retrotranscription
SCFAs: short-chain fatty acids
SNORD95: small nuclear RNA, C/D box 95
UC: ulcerative colitis
ZO: zonula occludens
